# Integrated multigene expression panel to prognosticate patients with gastric cancer

**DOI:** 10.18632/oncotarget.24661

**Published:** 2018-04-10

**Authors:** Mitsuro Kanda, Kenta Murotani, Haruyoshi Tanaka, Takashi Miwa, Shinichi Umeda, Chie Tanaka, Daisuke Kobayashi, Masamichi Hayashi, Norifumi Hattori, Masaya Suenaga, Suguru Yamada, Goro Nakayama, Michitaka Fujiwara, Yasuhiro Kodera

**Affiliations:** ^1^ Department of Gastroenterological Surgery (Surgery II), Nagoya University Graduate School of Medicine, Nagoya, Japan; ^2^ Clinical Research Center, Aichi Medical University Hospital, Nagakute, Japan

**Keywords:** gastric cancer, expression panel, prognosis, biomarker

## Abstract

Most of the proposed individual markers had limited clinical utility due to the inherent biological and genetic heterogeneity of gastric cancer. We aimed to build a new molecular-based model to predict prognosis in patients with gastric cancer. A total of 200 patients who underwent gastric resection for gastric cancer were divided into learning and validation cohorts using a table of random numbers in a 1:1 ratio. In the learning cohort, mRNA expression levels of 15 molecular markers in gastric tissues were analyzed and concordance index (C-index) values of all single and combinations of the 15 candidate markers for overall survival were calculated. The multigene expression panel was designed according to C-index values and the subpopulation index. Expression scores were determined with weighting according to the coefficient of each constituent. The reproducibility of the panel was evaluated in the validation cohort. C-index values of the 15 single candidate markers ranged from 0.506–0.653. Among 32,767 combinations, the optimal and balanced expression panel comprised four constituents (*MAGED2, SYT8, BTG1*, and *FAM46*) and the C-index value was 0.793. Using this panel, patients were provisionally categorized with scores of 1–3, and clearly stratified into favorable, intermediate, and poor overall survival groups. In the validation cohort, both overall and disease-free survival rates decreased incrementally with increasing expression scores. Multivariate analysis revealed that the expression score was an independent prognostic factor for overall survival after curative gastrectomy. We developed an integrated multigene expression panel that simply and accurately stratified risk of patients with gastric cancer.

## INTRODUCTION

Gastric cancer is still a severe public health problem worldwide, particularly in Eastern Asia [1]. While stage I gastric cancer may be curable by surgery alone, patients with advanced gastric cancer are at risk of death due to disease recurrence after initial tumor resection and failure to respond to subsequent chemotherapy [2, 3]. This underscores the importance of building a new risk stratification model for accurate prediction of prognosis, disease monitoring, and evaluation of treatment response.

Currently, endoscopy, and enhanced computed tomography are still the standard tests for diagnosing and staging gastric cancer [4, 5]. However, these are invasive procedures with a significant cost for patients. However, noninvasive serum tumor markers such as carcinoembryonic antigen (CEA) and carbohydrate antigen (CA) 19-9 are widely used in clinical practice, but have limited sensitivity and specificity, limiting their utility in decision making and management of patients with gastric cancer [6–8]. With the development of genomics, proteomics, and metabolomics, an increasing number of biomarkers have been identified and studied [9]. This holds promise that novel noninvasive markers with potential clinical value will be discovered to improve the management of gastric cancer [10]. However, due to the inherent heterogeneity of gastric cancer in terms of its biological and genetic characteristics, most individual markers have shown limited value in predicting differences in biology of the individual tumors and ultimately, in predicting clinical outcomes.

Recently, the concept of combining multiple markers has shifted the paradigm away from single gene analysis, providing more reliable insight into tumor biology, and yielding more robust oncological information. The Oncotype DX^®^ Colon Cancer Assay (Genomic Health, Redwood City, CA, USA), for example, utilizes a quantitative reverse transcription-polymerase chain reaction (RT-PCR)-based panel test using 12 molecular markers and has been validated in large clinical trials as a significant predictor of recurrence in stage II colon cancer [11]. It is a good example of success in demonstrating that comprehensive characterization of individual patients’ tumors is key to realizing the potential of personalized therapeutic strategies [12]. Still, there is room for improvement in assay simplification associated with a reduction in technical requirements, cost, and time. Taking into account the clinical application, an ideal assay strikes a balance between accuracy and simplicity.

These realities prompted us to build a predictive model for gastric cancer risk assessment. The aim of this study was to develop a simple and accurate integrated multigene expression panel that can provide clinical guidance in determining the optimal treatment for gastric cancer.

## RESULTS

### Development of an integrated multigene expression panel

**Figure 1:**
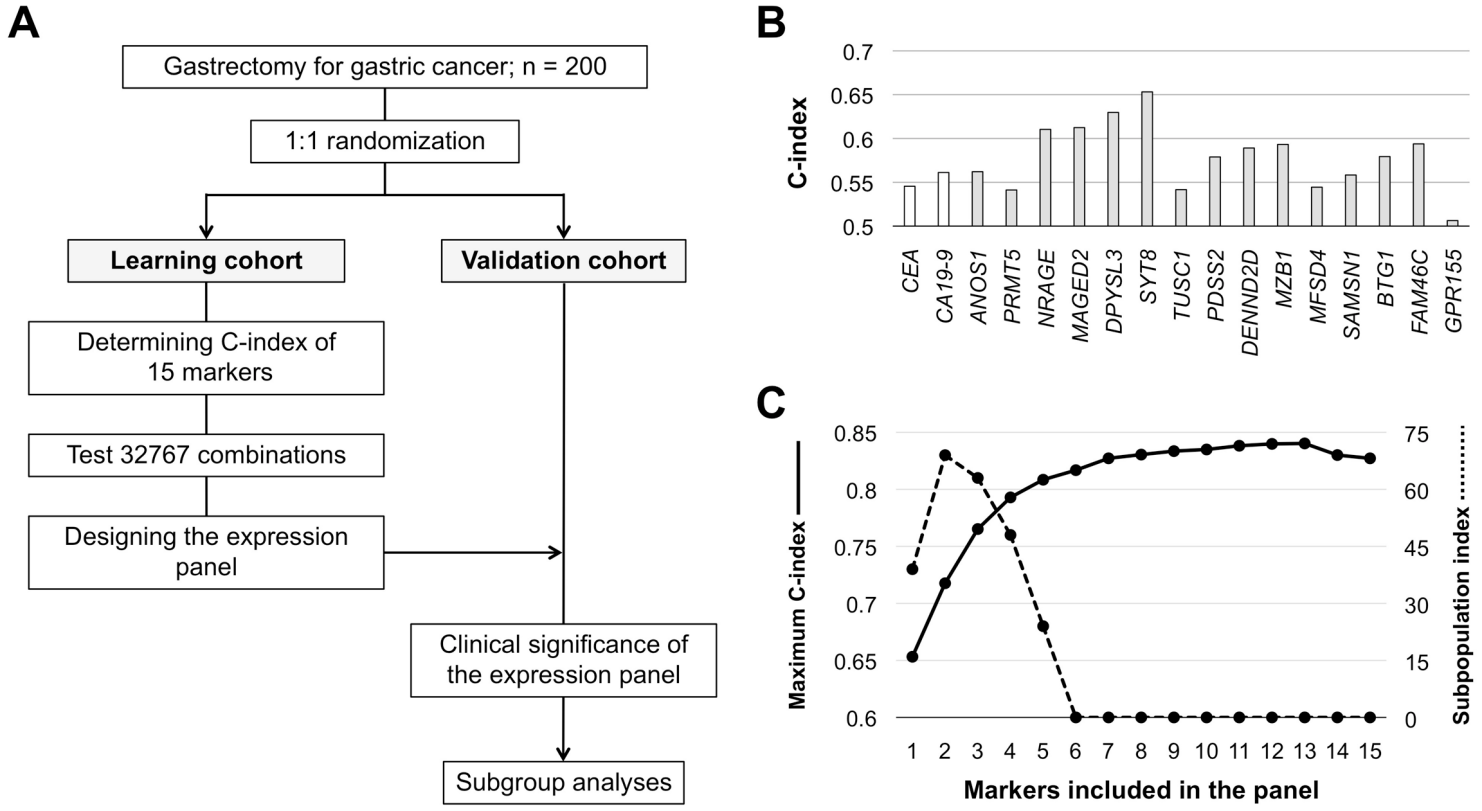
Development process of the integrated multigene expression panel **(A)** Flowchart. **(B)** C-index values of the 15 candidate molecular markers and preoperative serum CEA and CA19-9 levels. **(C)** C-index values and the subpopulation index according to the number of markers included in the panel.

**Figure 2:**
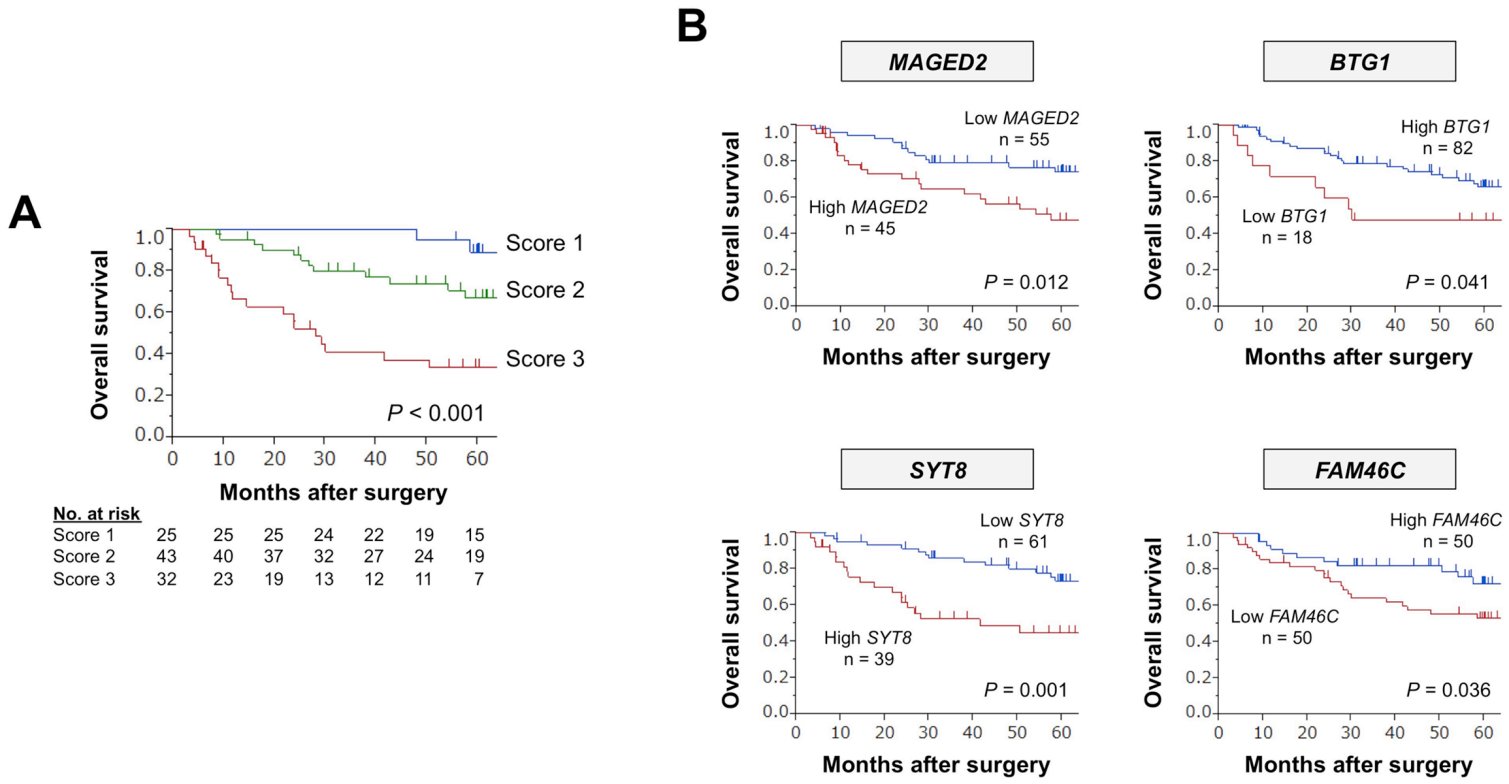
Predictive values of the expression panel and individual single markers in the learning cohort **(A)** Overall survival of patients with expression scores 1, 2, and 3. **(B)** The prognostic value of each constituent.

After randomized assignment of patients, there were no significant differences in patient characteristics between the learning and validation cohorts (Figure 1A and Supplementary Table 1). Concordance index (C-index) values of the 15 single candidate markers ranged from 0.506–0.653, and those of preoperative serum CEA (cutoff 5 ng/ml) and CA19-9 (cutoff 37 IU/ml) were 0.545 and 0.561, respectively (Figure 1B). C-index values of all single and combinations of the 15 candidate markers (neither CEA nor CA19-9 included) for overall survival were calculated and counted for 32,767 patterns. The highest C-index value among all combinations was 0.840, which was determined for the expression panel consisting of 13 markers (Figure 1C). The larger the number of markers included in the panel, the greater the number of subpopulations into which patients were clustered with a corresponding decrease in the minimal number of patients in a subpopulation. The subpopulation index rapidly decreased after the number of markers was ≥5 (Figure 1C). We decided that the optimal and balanced number of markers was four (C-index >0.75 and subpopulation index >45). The expression panel having the greatest C-index among combinations of four constituents comprised *MAGED2, SYT8, BTG1*, and *FAM46*, and the C-index value was 0.793 (Supplementary Table 2). The expression index was determined by weighting each marker using the coefficient, and then provisionally categorized into score 1 (expression index <40), score 2 (index 41–80), and score 3 (index ≥81). The scoring system clearly stratified patients into favorable, intermediate, and poor overall survival groups (Figure 2A), and none of the individual constituents of the expression panel (*MAGED2, SYT8, BTG1*, and *FAM46*) exhibited the equivalent stratifying performance compared with the multigene expression panel (Figure 2B). Based on these findings, the scoring system proceeded to the validation stage using another cohort.

### Clinical significance of the integrated multigene expression panel

**Figure 3:**
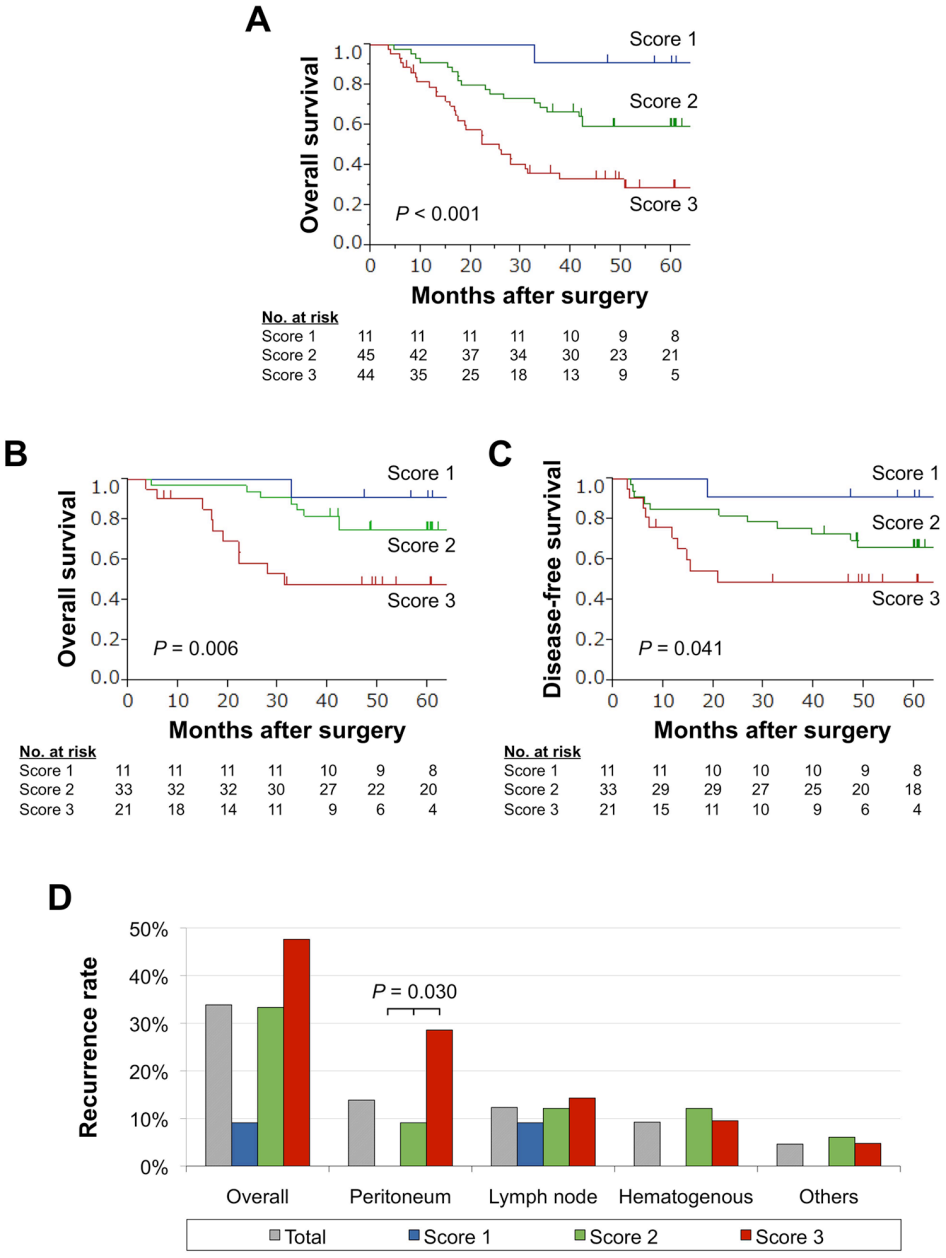
Prognostic impact of the expression panel in the validation cohort **(A)** Overall survival of patients with expression scores 1, 2, and 3. **(B)** Overall survival of patients with stage I-III gastric cancer. **(C)** Disease-free survival of patients with expression scores 1, 2, and 3 after curative gastrectomy. **(D)** Frequency of sites of initial recurrence for each expression score.

The reproducibility of the panel was evaluated in the validation cohort. The overall survival of patients with expression scores 1, 2, and 3 were clearly distinguished from each other (Figure 3A). No significant differences were found with respect to histology, tumor depth differentiation. In contrast, higher expression scores were significantly associated with larger tumor size, lymph node metastasis, peritoneal metastasis, hepatic metastasis, and advanced disease stage (Table 1).

**Table 1:** Association between expression scores and clinicopathological parameters in the validation set

Variables	Score 1 (n = 11)	Score 2 (n = 45)	Score 3 (n = 44)	*P*
Age				0.072
< 70 years	3	29	23	
≥ 70 years	8	16	21	
Sex				0.989
Male	8	32	31	
Female	3	13	13	
CEA (ng/ml)				0.085
≤ 5	10	37	29	
> 5	1	8	15	
CA19-9 (IU/ml)				0.136
≤ 37	10	37	30	
> 37	1	8	14	
Tumor location				
Entire	1	0	8	
Upper third	3	10	6	0.026
Middle third	4	13	10	
Lower third	3	22	20	
Tumor size (mm)				<0.001
< 50	9	12	8	
≥ 50	2	33	36	
Tumor multiplicity				0.485
Solitary	11	42	41	
Multiple	0	3	3	
Tumor depth (UICC)				0.058
pT1	4	8	2	
pT2	0	4	2	
pT3	1	8	14	
pT4	6	25	26	
Histology				0.157
Well differentiated	1	5	0	
Moderately differentiated	3	13	13	
Poorly differentiated	7	24	29	
Signet ring cell	0	2	0	
Mucinous	0	1	2	
Differentiation				0.581
Differentiated	4	18	13	
Undifferentiated	7	27	31	
Lymphatic involvement				0.197
Absent	3	6	3	
Present	8	39	41	
Vascular invasion				0.063
Absent	6	22	12	
Present	5	23	32	
Infiltrative growth type				0.177
Invasive growth	2	18	21	
Expansive growth	9	27	23	
Lymph node metastasis				
Absent	5	19	4	<0.001
Present	6	26	40	
Peritoneal metastasis				0.013
Absent	11	33	28	
Present	0	12	16	
Synchronous hepatic metastasis				
Absent	11	45	39	0.014
Present	0	0	5	
UICC stage				<0.001
I	4	11	1	
II	2	7	6	
III	5	15	14	
IV	0	12	23	

CEA, carcinoembryonic antigen; CA19-9, carbohydrate antigen 19-9; UICC, Union for International Cancer Control.

When focused on patients who underwent curative gastrectomy (stage I–III gastric cancer), overall (Figure 3B) and disease-free survival rates (Figure 3C) gradually decreased with increasing expression score. Multivariable analysis revealed that expression score 3 was an independent prognostic factor for overall survival after curative gastrectomy (hazard ratio 3.18, 95% confidence interval 1.19–8.62, *P* = 0.021; Table 2). Overall recurrence rates and frequency of each recurrent pattern observed according to the expression score are depicted in Figure 3D. No patients with the score 1 experienced peritoneal and/or hepatic recurrences. In contrast, the prevalence of peritoneal recurrences showed a stepwise increase in proportion to the expression score (Figure 3D).

**Table 2:** Prognostic factors for overall survival in patients who underwent curative resection in the validation cohort

Variables	n	Univariate			Multivariate		
		Hazard ratio	95% CI	P	Hazard ratio	95% CI	P
Age (≥ 70)	27	0.81	0.30–1.99	0.656			
Gender (male)	20	1.36	0.53–4.19	0.541			
CEA (> 5 ng/ml)	11	1.34	0.38–3.66	0.611			
CA19-9 (> 37 IU/ml)	11	2.85	1.00–7.14	0.049	1.60	0.54–4.30	0.379
Tumor location (lower third)	33	0.81	0.32–1.98	0.652			
Tumor size (≥ 50 mm)	38	2.35	0.91–7.23	0.079			
Tumor depth (pT4, UICC)	28	2.15	0.89–5.49	0.089			
Tumor differentiation (undifferentiated)	39	1.38	0.56–3.67	0.488			
Lymphatic involvement	55	4.10	0.85–73.6	0.087			
Vascular invasion	35	6.00	2.01–25.7	<0.001	4.31	1.33–19.3	0.013
Invasive growth	20	1.99	0.80–4.82	0.133			
Lymph node metastasis	39	4.96	1.66–21.3	0.003	1.06	0.19–4.77	0.939
UICC stage (III)	34	4.59	1.68–16.1	0.002	3.66	1.08–15.6	0.037
Expression score (3)	21	3.68	1.49–9.12	0.005	3.18	1.19–8.62	0.021

CEA, carcinoembryonic antigen; CA19-9, carbohydrate antigen 19-9; UICC, Union for International Cancer Control.

## DISCUSSION

In this study, we analyzed 32,767 patterns and built a new prognostic model, an integrated multigene expression panel that can clearly stratify patients into low, intermediate, and high risk after gastrectomy for gastric cancer. The advantages of the panel are manifested in the following ways: a novel panel comprising original molecular markers, results presented using a simple scoring system, high predictive value with respect to overall and disease-free survival, and, confirmed reproducibility as demonstrated in both the learning and validation cohorts.

A growing body of evidence has demonstrated that gastric cancer is a complex and heterogeneous disease with substantial variation in its molecular and clinical characteristics [13, 14]. Since it is unlikely that a single molecular marker can faithfully represent the various oncological signatures, more reliable and convenient prognostic models are required to enhance the long-term survival of patients with gastric cancer [9, 15]. Combining multiple independently predictive markers has been demonstrated to improve accuracy in large clinical trials for breast, prostate, and colorectal cancer; however, few studies have investigated the diagnostic efficacy of three-dimensional combined biomarkers for gastric cancer [16–18].

Given that our aim was to develop a simple and high-performance multigene expression panel, certain procedures were required to be followed. The larger the number of markers included in the panel, the greater the number of subpopulations into which patients were clustered with a corresponding decrease in the minimal number of patients in any given subpopulation. The subpopulation index was used to optimize the number of markers included in the panel, and inclusion of four markers was found to be the most objectively balanced system. To maximize performance of the expression panel, a weighting using the coefficient of each constituent was employed to determine the expression index for all patients [19]. Thereafter, patients were stratified based on their expression score (1 to 3) according to the expression index, which was a more straightforward patient stratification method compared with using continuous numeric variables. Considering that our attempt was certainly exploratory, the validation process was necessary to evaluate the validity of the procedure used in the development of the multigene expression panel. As a result, the predictive value of the panel was reproduced successfully in the validation set. The consistent findings give us confidence that the expression panel may have potential in the risk stratification of patients with gastric cancer.

The strength of the final panel is that candidate biomarkers used for the development of the panel have been previously validated biologically. The four constituents of the expression panel, *MAGED2, SYT8, BTG1*, and *FAM46*, have individual roles in gastric cancer progression. *MAGED2* is a cell adherent molecule and belongs to the melanoma-associated antigen family, which plays important roles in cancer development, progression, and resistance to treatment [20, 21]. We reported that increased levels of tissue and serum *MAGED2* were associated with distant metastasis in gastric cancer [22]. *SYT8* encodes a single-pass membrane protein involved in membrane trafficking [23]. Elevated *SYT8* levels were significantly and specifically associated with peritoneal metastasis, and intraperitoneal administration of an *SYT8*-specific small interfering RNA inhibited the growth of peritoneal nodules and prolonged survival in mouse xenograft models [24]. *BTG1* reportedly is a mediator of B-cell differentiation and may act as a tumor suppressor because of its inhibitory effects on proliferation and cell cycle progression [25, 26]. In our previous study, downregulation of *BTG1* was associated with larger tumor size and lymph node metastasis [27]. *FAM46C* is a signal transducer that stabilizes mRNA and is frequently mutated and downregulated in gastric cancer tissues [28]. We found that downregulation of *FAM46C* served as a predictive marker of hepatic recurrence after curative gastrectomy [29]. Since four different types of biomarkers have distinguishing features and contribute to metastatic patterns, they complementarily interacted with each other and contributed to the expression panel, having an improved predictive performance in gastric cancer, even though it is an extremely heterogeneous disease. Furthermore, use of our study concept can leverage current knowledge of single molecular markers and bring them to the next stage, which would be an important step forward in the realization of precision medicine.

To translate results of the present study to the clinic will involve discussion about how best to use the expression panel. Our findings highlight that the integrated multigene expression panel enables physicians to easily identify individuals expected to have an excellent prognosis (low risk), and conversely those expected to have an adverse outcome (high risk). For patients at low risk, avoidance of excessive intervention both in disease monitoring and treatment can reduce the burden for patients, as well as medical costs. In contrast, intensive systemic surveillance including enhanced computed tomography to detect signs of peritoneal, nodal or hepatic recurrences, and aggressive adjuvant therapy could be considered for patients at high risk. For patients at intermediate risk, standard management conformable to the treatment guidelines is recommended [30]. Patients who underwent curative gastrectomy are recognized as a delicate population characterized by varied prognosis (range from complete cure to early recurrence) that will likely benefit from accurate risk stratification. Therefore, for patents with resectable gastric cancer, the expression panel might merit inclusion as an adjustment factor or one of the endpoints in prospective clinical trials evaluating survival benefit of systemic adjuvant chemotherapy in gastric cancer [31]. In this study, expression levels were determined using surgically-resected gastric tissues. Since endoscopic biopsy samples are also available for mRNA analysis, expression scores can be determined before surgery and may contribute to decision-making regarding indication of perioperative treatment or surgery. Because the clinical utility of the expression panel to accurately predict patient outcomes is the ultimate goal, the present work should be viewed as an important first step but not as the definitive answer.

This study had some limitations. Despite an effort to reduce selection bias using a 2-step evaluation, the retrospective nature of the study, the relatively small cohort size, the usage of some old samples, and the long period of study may have biased the data. Although we designed a 2-step evaluation protocol of the predictive value of our integrated multigene expression panel, extra-validation and a prospective large-scale observational study will be required for the next step toward translation to the clinical practice. Although mRNA expression levels were used because they are easy to quantify objectively, the use of IHC could be considered given that it is a readily accessible and commonly used technique in clinical practice.

Taken together, we developed an integrated multigene expression panel for patients with gastric cancer that may maximize the predictive performance of each single marker, enable accurate risk stratification, and eventually contribute to personalized medicine in the field of surgical oncology.

## MATERIALS AND METHODS

### Patients, sample collection, and randomization

Primary gastric cancer tissues and corresponding noncancerous adjacent tissues were collected from 200 gastric cancer patients who underwent gastric resection without preoperative treatment at the Department of Gastroenterological Surgery, Nagoya University Hospital between 2001 and 2014. Tissue samples were collected, frozen immediately in liquid nitrogen, and stored at –80°C until used for RNA extraction (average 28 days). RNA was extracted from tumor samples with approximately 5 mm diameters that did not contain a necrotic component. Using a table of random numbers, 200 patients were divided into learning (n = 100) and validation cohorts (n = 100) in a 1:1 ratio. Markers to be included in the integrated expression panel were determined using the learning cohort, and the clinical predictive performance of the panel was subsequently evaluated in the validation cohort. This study conformed to the ethical guidelines of the Declaration of Helsinki and was approved by the Institutional Review Board of Nagoya University, Japan (approval number 2014-0043). Written informed consent for use of clinical samples and data, as required by the institutional review board, was obtained from all patients.

### Molecular markers comprising the expression panel

From our recently published papers, 15 molecular markers were evaluated as candidate biomarkers (Table 3): anosmin-1 (*ANOS1*), protein arginine methyltransferase 5 (*PRMT5*), neurotrophin receptor-interacting melanoma antigen-encoding protein (*NRAGE*), melanoma antigen gene family member D2 (*MAGED2*), dihydropyrimidinase like 3 (*DPYSL3*), synaptotagmin VIII (*SYT8*), tumor suppressor candidate 1 (*TUSC1*), decaprenyl diphosphate synthase subunit 2 (*PDSS2*), DENN domain containing 2D (*DENND2D*), marginal zone B and B1 cell-specific protein (*MZB1*), major facilitator superfamily domain containing 4 (*MFSD4*), SAM domain, SH3 domain and nuclear localization signals 1 (*SAMSN1*), BTG anti-proliferation factor 1 (*BTG1*), family with sequence similarity 46, member C (*FAM46C*), and G protein-coupled receptor 155 (*GPR155*). Quantitative real-time RT-PCR (qRT-PCR) was performed to determine mRNA expression levels, as described previously [22, 24, 27, 29, 32-42]. Sequences of specific primers are listed in Supplementary Table 3. Patients were categorized into the two groups using cut-off values from previous studies (Table 3).

**Table 3:** List of candidate markers aberrantly expressed in gastric cancer

Symbol	Name	Location	Function	Status in GC^*^	Cutoff^*^
ANOS1	anosmin-1	Xp22.31	Neural cell adhesion and axonal migration	Upregulated	C median
PRMT5	protein arginine methyltransferase 5	14q11.2	Transcriptional regulation, and the assembly of small nuclear ribonucleoproteins	Upregulated	C median
NRAGE	neurotrophin receptor-interacting melanoma antigen-encoding protein	Xp11.22	Pro-apoptotic factor required for the normal developmental apoptosis	Upregulated	C mean
MAGED2	MAGE family member D2	Xp11.21	Tumor specific antigens	Upregulated	C/N >1
DPYSL3	dihydropyrimidinase like 3	5q32	Cell-adhesion factor	Upregulated	C median
SYT8	synaptotagmin VIII	11p15.5	Membrane trafficking protein	Upregulated	C 0.005
TUSC1	tumor suppressor candidate 1	9p21.2	Unknown	Downregulated Hypermethylated	C 1st quartile
PDSS2	decaprenyl diphosphate synthase subunit 2	6q21	Synthesis of coenzyme Q10	Downregulated Hypermethylated	C/N <0.5
DENND2D	DENN domain containing 2D	1p13.3	Membrane trafficking protein regulating Rab GTPases	Downregulated Hypermethylated	C/N <0.5
MZB1	marginal zone B and B1 cell-specific protein	5q31.2	B cell activation	Downregulated Hypermethylated	C median
MFSD4	major facilitator superfamily domain containing 4	1q32.1	Membrane transporter	Downregulated Hypermethylated	C = 0.006
SAMSN1	SAM domain, SH3 domain and nuclear localization signals 1	21q11.2	Cytoplasmic adaptor protein	Downregulated	C median
BTG1	BTG anti-proliferation factor 1	12q21.33	Regulates cell growth and differentiation	Downregulated	C/N < 1/3
FAM46C	family with sequence similarity 46, member C	1p12	Transcription factor	Downregulated	C median
GPR155	G protein-coupled receptor 155	2q31.1	Mediator of the visual sensing, immune function, and cell proliferation	Downregulated	C 0.0009

^*^From references 22, 24, 27, 29 and 32-42. C, expression levels in cancerous tissue; N, expression levels in adjacent non-cancerous tissue.

### Development and validation of the integrated multigene expression panel

To build an integrated multigene expression panel, the following processes were carried out in the learning cohort. The study flowchart is shown in Figure 1A. First, C-index values of all single and combinations of the 15 candidate markers for overall survival were calculated. Second, the best C-index values for each number of combinations (1–15) were calculated. The larger the number of markers included in the panel, the greater the number of subpopulations that patients were clustered into with a corresponding decrease in the minimal number of patients in a subpopulation. Thus, third, we used the subpopulation index, calculated as number of constituents × the minimal patient number in a subpopulation, for each number of combinations to determine the most well-balanced number of markers to be included in the expression panel [19]. Fourth, the expression index was determined with weighting according to the coefficient in a Cox regression of each constituent. Fifth, provisional cutoff for the scoring (score 1 to 3) were determined in the discovery set based on the following concept. The lower cutoff line was set strictly to achieve careful selection of patients with excellent postoperative outcomes, even if the population becomes small. Similarly, the upper cutoff line was set to select patients at very high risk. Sixth, patients were classified as having expression scores of 1, 2, and 3 according to the cutoff lines of expression index. Last, the reproducibility of the integrated multigene expression panel was evaluated in the validation cohort.

### Statistical analysis

The qualitative χ^2^ and quantitative Mann-Whitney tests were used to compare the two groups. Survival rates were calculated using the Kaplan-Meier method, and the difference between curves was analyzed using the log-rank test. The Cox regression model was used to evaluate the overall survival hazard ratio associated with each variable. The prediction score was internally validated using the C-index. The C-index is a probability of concordance between predicted and observed survival, with C = 0.5 for random predictions and C = 1 for a perfectly discriminating score. The C-index was evaluated on the discovery set using bootstrapping with 10,000 resamples [43]. Statistical analysis was performed using JMP 10 software and SAS9.4 (SAS Institute Inc., Cary, NC, USA). *P* <0.05 indicates a statistically significant difference.

## SUPPLEMENTARY MATERIALS TABLES


