# Supplementary material for: Integrated multigene expression panel to prognosticate patients with gastric cancer
Source: Oncotarget. 2018 Apr 10;9(27):18775–85. doi: 10.18632/oncotarget.24661 (PMC5922354; doi:10.18632/oncotarget.24661)
Supplement: Supplementary file 1 [file oncotarget-09-18775-s001.pdf]

# Integrated multigene expression panel to prognosticate patients with gastric cancer

## SUPPLEMENTARY MATERIALS

**Supplementary Table 1: Characteristics of patients in the learning and validation cohorts**

| | Learning set (n=100) | Validation set (n=100) | P |
|---|---|---|---|
| Age (years), mean ± SD | 65.6 ± 11.5 | 65.7 ± 11.0 | 0.955 |
| Sex (male/female) | 70 / 30 | 71 / 29 | 0.877 |
| Tumor location | | | |
| Entire | 9 | 9 | |
| Upper third | 23 | 19 | 0.613 |
| Middle third | 32 | 27 | |
| Lower third | 36 | 45 | |
| Macroscopic type | 20 | 15 | |
| Borrmann type 4/5 | 20 | 15 | 0.351 |
| Others | 80 | 85 | |
| UICC stage | | | |
| I | 18 | 15 | |
| II | 18 | 15 | 0.805 |
| III | 28 | 34 | |
| IV | 36 | 35 | |
| Follow up months, median | 61.9 | 60.9 | 0.617 |

SD, standard deviation.

**Supplementary Table 2: Proposed expression panel to predict overall survival**

| C-index; expression panel | Symbol | C-index; single marker | Coefficient | HR | 95% CI | P |
|---|---|---|---|---|---|---|
| 0.793 | MAGED2 | 0.612 | 1.484 | 4.412 | 1.847-10.54 | <0.001 |
| | SYT8 | 0.653 | 1.407 | 4.085 | 1.997-8.358 | <0.001 |
| | BTG1 | 0.579 | 1.817 | 6.156 | 2.246-16.88 | <0.001 |
| | FAM46C | 0.594 | 0.842 | 2.321 | 1.120-4.810 | 0.023 |

HR, hazard ratio; CI, confidence interval.

**Supplementary Table 3: Specific primers used for quantitative RT-PCR**

| Gene | Type | Sequence (5´ - 3´) | Product size |
|---|---|---|---|
| ANOS1 | forward | AACAATGGTTCCCTGGTTTG | 110 bp |
| | reverse | TCACAAAAGCTTTGGCACTG | |
| PRMT5 | forward | TCTCATGGTTTCCCATCCTC | 102 bp |
| | reverse | CCTTCTTGGAATTGCTGCAT | |
| NRAGE | forward | GATTCCCTCAGACCTTTGC | 170 bp |
| | reverse | GAAGGAATCTGAGGCTTCAG | |
| MAGED2 | forward | TAGAGAAGGCAGACGCATCC | 110 bp |
| | reverse | AAGCGAGTTAGACCTGCACC | |
| DPYSL3 | forward | AGAAGAAGGAGGGAGGGAGC | 110 bp |
| | reverse | CTCCCTTGATAAGGAGACGG | |
| SYT8 | forward | GCTTCTCTCTCCGGTACGTG | 196 bp |
| | reverse | AGGAAGGTGAAGGCCTCATT | |
| TUSC1 | forward | ACATGTACAGTTCCCCTGCC | 110 bp |
| | reverse | GTGTTTCTTGGCACCCAGTT | |
| PDSS2 | forward | GAATCAGGTAGTGTCAGAGG | 181 bp |
| | reverse | GAGGCTATTCCAGCTGTCATG | |
| DENND2D | forward | CACTGCTCTACCCCTTCAGC | 204 bp |
| | reverse | TTTTTCATCACCAACCGACA | |
| MZB1 | forward | CTCACAGGCCCAGGACTTAG | 219 bp |
| | reverse | TGTGGCTGACACCTTCTCTG | |
| MFSD4 | forward | CAACATGCAGCTGGTAAGGA | 192 bp |
| | reverse | ACCCTGGAGACATGGAACAG | |
| SAMSN1 | forward | TGCTCAAGAGAAAGCCATCC | 97 bp |
| | reverse | TTATTCCGAAAACGATCGAAA | |
| BTG1 | forward | CTGCAGACCTTCAGCCAGA | 104 bp |
| | reverse | CGAATACAACGGTAACCCGA | |
| FAM46C | forward | CATGTGGCTCTTCCAACAGA | 219 bp |
| | reverse | CTTCAGCTCCACGTTCTTCC | |
| GPR155 | forward | AGCAAAGCTGGACTATTCCCT | 125 bp |
| | reverse | GCCACCAAATAAATGTACTGGA | |
| GAPDH | forward | GAAGGTGAAGGTCGGAGTC | 226 bp |
| | probe | CAAGCTTCCCGTTCTCAGCC | |
| | reverse | GAAGATGGTGATGGGATTTC | |
